# Root-associated fungi of *Vaccinium carlesii* in subtropical forests of China: intra- and inter-annual variability and impacts of human disturbances

**DOI:** 10.1038/srep22399

**Published:** 2016-03-01

**Authors:** Yanhua Zhang, Jian Ni, Fangping Tang, Kequan Pei, Yiqi Luo, Lifen Jiang, Lifu Sun, Yu Liang

**Affiliations:** 1 College of Life Sciences, Shaoxing University, Shaoxing, China; 2 State Key Laboratory of Vegetation and Environmental Change, Institute of Botany, Chinese Academy of Sciences, Beijing, China; 3 Department of Microbiology and Plant Biology, University of Oklahoma, Norman, OK 73019, USA

## Abstract

Ericoid mycorrhiza (ERM) are expected to facilitate establishment of ericaceous plants in harsh habitats. However, diversity and driving factors of the root-associated fungi of ericaceous plants are poorly understood. In this study, hair-root samples of *Vaccinium carlesii* were taken from four forest types: old growth forests (OGF), secondary forests with once or twice cutting (SEC I and SEC II), and *Cunninghamia lanceolata* plantation (PLF). Fungal communities were determined using high-throughput sequencing, and impacts of human disturbances and the intra- and inter-annual variability of root-associated fungal community were evaluated. Diverse fungal taxa were observed and our results showed that (1) Intra- and inter-annual changes in root-associated fungal community were found, and the Basidiomycota to Ascomycota ratio was related to mean temperature of the sampling month; (2) Human disturbances significantly affected structure of root-associated fungal community of *V. carlesii*, and two secondary forest types were similar in root-associated fungal community and were closer to that of the old growth forest; (3) Plant community composition, edaphic parameters, and geographic factors significantly affected root-associated fungal communities of *V. carlesii*. These results may be helpful in better understanding the maintenance mechanisms of fungal diversity associated with hair roots of ERM plants under human disturbances.

Plants of Ericaceae distribute all over the world, and are common species especially in habitats of heathlands, tundra, and forests^1^. Plant species from Ericaceae could form ericoid mycorrhiza (ERM), which are expected to help them to establish successfully in habitats with low nutrients, acid soils, cold or drought stresses, and heavy metal pollutions^2^,^3^,^4^,^5^,^6^. However, mycorrhizal and other fungi associated with their hosts were varied along the environment and temporal heterogeneity^7^,^8^,^9^,^10^,^11^,^12^.

Significant seasonal dynamics have been observed for soil fungi^13^,^14^,^15^, ectomycorrhizal (ECM) fungi^16^,^17^, and arbuscular mycorrhizal (AM) fungi^18^,^19^ in different ecosystems. Seasonal changes of ERM fungi were also found in typical Mediterranean climate regions of Australia^20^,^21^,^22^. Superiority of hair roots with ERM and their endophytes were observed in colder and more humid months^21^, and fungal richness and phylogenetic diversity was also found having the same trends under similar conditions^23^. Lentendu *et al.*^24^ suggested that soil moisture may be an important factor in determining the seasonal patterns of ERM fungi. Seasonal changes in environmental factors such as temperature, precipitation, and nutrient availability may be related to seasonal variations of fungal communities^24^.

Fungal community changes have been observed not only in seasonality but also in inter-annual variations^25^. These inter-annual variations may be related to both succession of plant communities and inter-annual climatic factors^7^,^10^,^11^,^15^,^26^,^27^,^28^. de Román and de Miguel^16^ detected that percentage of ECM colonization significantly increased over 3-years period post fire, and long-term succession in microbial communities were also found in some chronosequence studies along successional gradients^7^,^10^,^11^,^27^,^28^. Inter-annual temporal dynamics were also observed by Cotton *et al.*^26^ in a 5-years study on AM fungal communities, in which 42% variations in AM fungal communities could be explained by the sampling year. However, the fluctuations of ERM fungal communities were less known.

Natural succession, land use changes and land management may change microclimate, edaphic and biotic factors, which would further influence diversity and composition of root-associated fungal communities^7^,^8^,^9^,^10^,^11^,^12^,^29^. For example, with the increase of stand ages, not only fungal communities but mycorrhizal types were distinct during succession^27^,^30^. Different mycorrhizal and soil fungi, such as AMF in a tropical dry ecosystem (primary forests, secondary forests and pastures) in Mexico^31^, ERM fungi in peatland sites (bog, rough grazing and forest plantation) in Ireland^12^, and soil fungi in southeast Asian tropical forests (original forests, secondary forests and oil palm agriculture)^32^, were investigated and the results suggested that human-activity-induced changes in plant community and abiotic environments were very important in shaping fungal community compositions at the landscape scale.

To evaluate the impacts of human disturbances on root-associated fungal communities of ericaceous plants, four forest types with different human disturbances were selected: old growth forests (OGF), secondary forests with once (SEC I) and twice (SEC II) disturbances, and *Cunninghamia lanceolata* plantations (PLF). Hair roots of *V. carlesii*, a common understory ericaceous plant species in subtropical forests of China, were collected in three years (2012–2014) and root-associated fungal communities were determined using high-throughput sequencing. Fungal diversity and community composition were compared between seasons, years and different human disturbances. Three hypothesis were proposed: (1) fungal community composition has intra- and inter-annual variations, and different fungal taxa may have different temporal patterns; (2) fungal composition and community dynamic associated with hair roots of *V. carlesii* would be influenced by different human disturbances, the stronger disturbance, the larger effects on fungi; (3) plant community composition, edaphic parameters, and geographic factors may affect the composition of fungal community in roots of *V. carlesii*.

## Results

### Changes of *Vaccinium carlesii* population and environmental factors along with disturbance gradient

As a common understory species in subtropical forests, *V. carlesii* was found in all four forest types along the disturbance gradient, e.g., old growth forests (OGF, without human disturbance at least 100 years), secondary forests with once clear-cut (SECI, about 50 years ago) and twice cut (SECII, clear-cut about 50 years ago, selected cut about 20 years ago), and *Cunninghamia lanceolata* plantation (PLF, clear-cut and planted *Cunninghamia lanceolata* about 20 years ago) (Fig. S1). Diameter at breast height (DBH) was much higher in OGF and individual density of *V. carlesii* was significantly higher in two secondary forests than those of OGF and PLF (Fig. S1). Soil properties in all forests were shown in Table S1. Soil pH was not significantly different in all forests, ranging from 4.73 to 4.77, a typical acid soil. Soil nutrient analysis showed that soil organic carbon (SOC), soil total nitrogen (STN), NO_3_^−^-N and available phosphorus (AP) were, but soil total phosphorus (STP) and NH_4_^+^-N were not significantly different along the disturbance gradient, indicating that more available nutrients appeared in OGF, and no different nutrient supply between disturbed forest types.

### Fungal diversity in hair roots of *V. carlesii*

**Figure 1:**
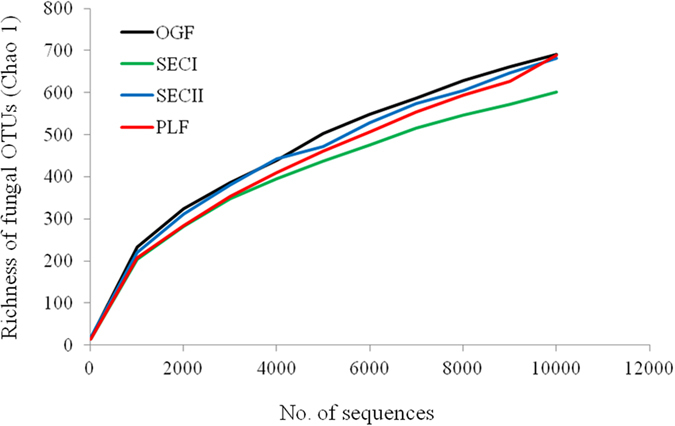
Rarefaction curves of fungal OTUs (by Chao1 estimates) in roots of *V. carlesii* in forests with different human disturbances, i.e. old growth forest (OGF), secondary forest I (SECI), secondary forest II (SECII), and plantation (PLF).

**Figure 2:**
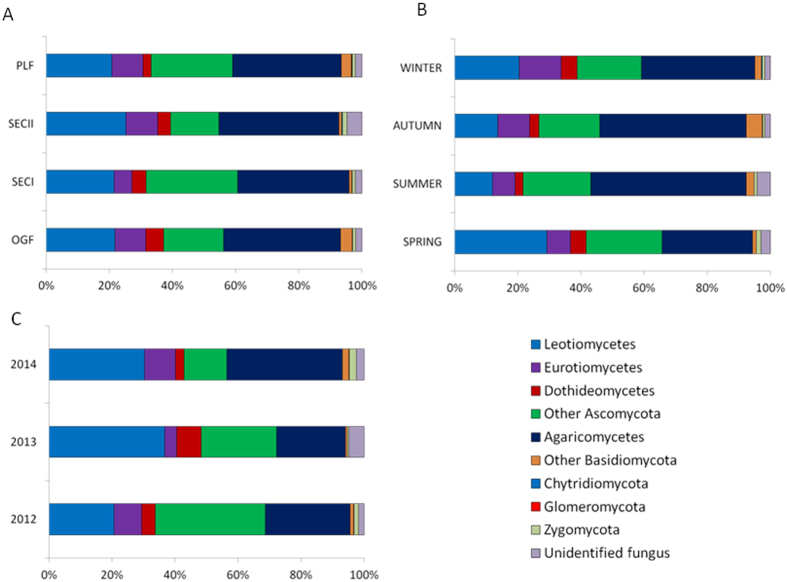
Proportions of fungal phyla and common fungal classes (>1%) within Ascomycota and Basidiomycota in different forest types, seasons, and years (only samples taken in spring were compared).

Rarefaction curves of fungal OTUs in roots of *V. carlesii* were shown in Fig. 1. While the mean number of OTUs in SECI was slightly lower, no significant differences were found between four forest types. There were 5595 OTUs in hair roots of *V. carlesii* in all forest types, including Ascomycota, Basidiomycota, Zygomycota, Chytridiomycota, and Glomeromycota (Fig. 2). Ascomycota and Basidiomycota were two dominant phyla in all forest types, and proportions of Zygomycota, Chytridiomycota, and Glomeromycota were only 1.14%, 0.08%, and 0.03%, respectively. Common classes included Leotiomycetes, Eurotiomycetes, Dothideomycetes, Sordariomycetes from Ascomycota and Agaricomycetes from Basidiomycota. Dominant fungal orders (>5%) included typical ERM orders such as Helotiales (21.9%), Sebacinales (8.9%), and Chaetothyriales (6.1%), as well as typical ECM orders such as Thelephorales (9.0%) and Russulales (8.9%).

**Figure 3:**
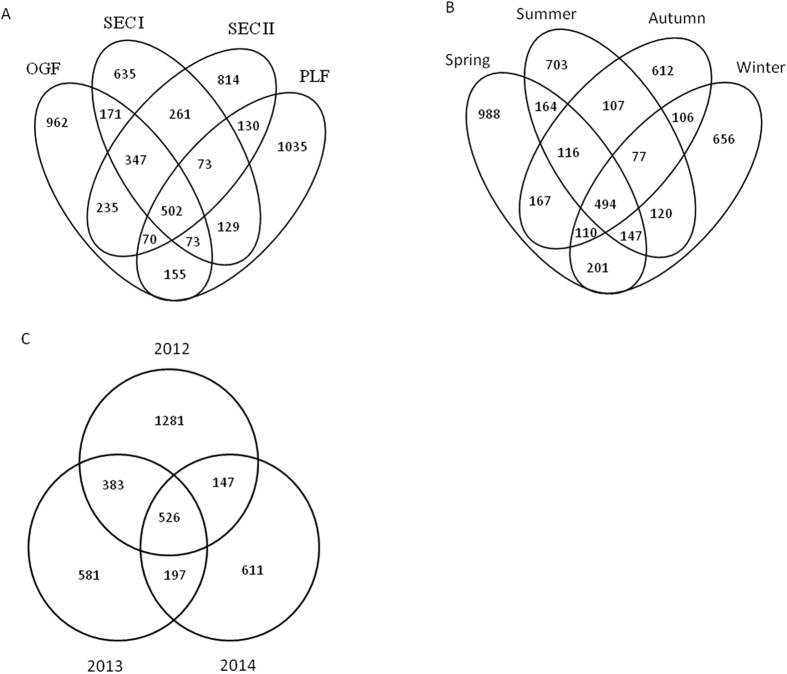
Venn diagram showing specific and shared OTUs of different forest types (A), seasons (B), and years (C) (only samples taken in spring were compared).

Venn diagrams showed the number of specific and shared OTUs of *V. carlesii* by seasons (Fig. 3B), years (Fig. 3C) and different human disturbances (Fig. 3A). Figure 3(B) showed that 494 OTUs was shared among four seasons in 2012, which was 10.36% of total 4768 OTUs. The seasonal specific OTUs in spring, summer, autumn and winter of 2012 were 988, 703, 612 and 656, accounting for 20.72%, 14.74%, 12.84% and 13.76% of total OTUs, respectively. Both specific and total OTU number in spring was much higher than those in the other three seasons.

In Fig. 3(C), of the total 3726 OTUs that were observed in spring of three years, only 526 OTUs were found in all three year, accounting for 87.1% of total reads in each sample. The specific OTUs occurred only in 2012 were much more than those in 2013 and 2014.

Total 2518, 2170, 2191, and 2435 OTUs were found in OGF, SECI, SECII, and PLF (Fig. 3A), of which 962, 653, 814, and 1035 OTUs were specific to these four forest types, respectively. 502 OTUs were shared by all forest types, accounting for 8.9% of the total OTU number. When excluding 502 OTUs shared by all four forest types, only 73 OTUs were found in both PLF and two secondary forests, which was much less than the shared OTUs between OGF and the secondary forests (347 OTUs).

### Fungal community composition varied with season and year and responses to human disturbance

**Figure 4:**
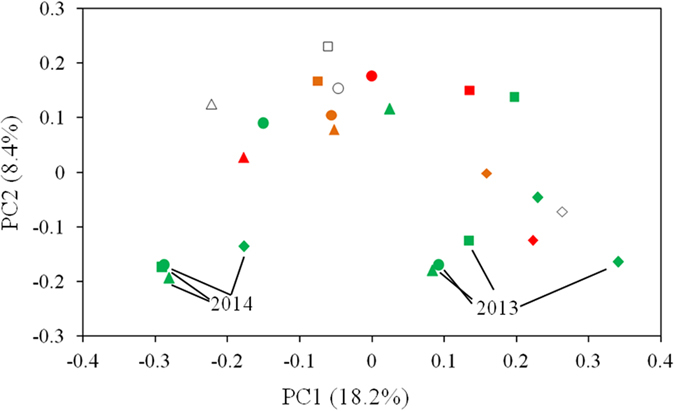
Principal Component Analysis (PCA) of root-associated fungi of *V. carlesii*. Forest types are shown as different shapes (OGF circle, SEC I square, SEC II triangle, PLF diamond), and seasons are shown as different colors (Spring green, Summer red, Autumn brown, Winter white).

**Figure 5:**
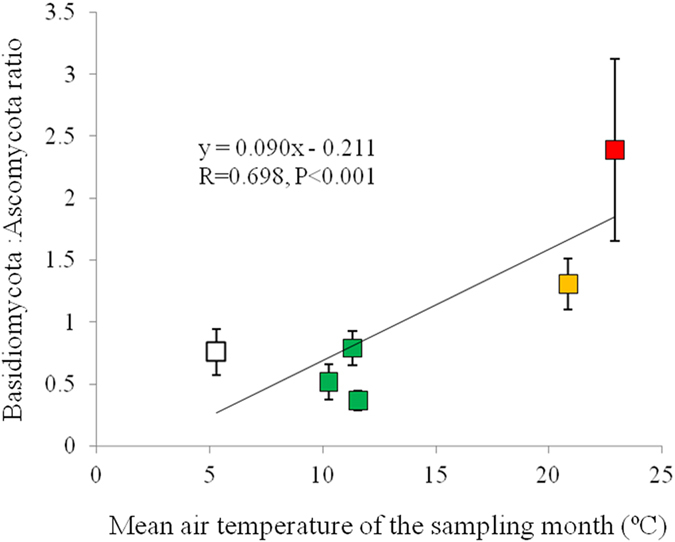
Relationship between mean air temperature of the sampling month and ratio of Basidiomycota to Ascomycotain root-associated fungal communities. Sampling season ⬜ winter, 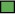 spring, 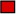 summer, 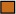 autumn.

Principal Component Analysis (PCA) results on community structure of root-associated fungi of *V. carlesii* in different forests sampled in different seasons of 2012 and spring of 2013 and 2014 were shown in Figs 4 and S2. Four seasons were not well separated in 2012 and root-associated fungal communities in spring of 2013 and 2014 were much distinct to those of 2012. The results of dominant phyla and classes showed that cold (spring and winter) and warm (summer and autumn) seasons had different compositions at high taxa level (Fig. 2B). The ratio of Basidiomycota to Ascomycota in root-associated fungal communities was significantly higher in summer and autumn and significantly correlated with mean air temperature of the sampling month (r = 0.698, P < 0.001, Fig. 5).

The results of PCA showed that fungal community structures of two secondary forests were more similar to old growth forests than to plantations for all six sampling dates (Figs 4 and S3).

### Indicator fungal species for seasons, years and forest types

There were 27 OTUs that showed significant preference to a specific season (Table 1), including 15, 2, 8, and 2 indicator species for spring, summer, autumn and winter, respectively. Among the indicator species for seasons, Helotiales 3, Helotiales 4, Helotiales 5, Dermateaceae 1, Herpotrichiellaceae 4 and Herpotrichiellaceae 5 are putative ERM fungi^1^,^22^,^33^,^34^,^35^; and Thelephoraceae (Thelephoraceae 1 and Thelephoraceae 2) are usually considered as members of ECM fungal family^36^.

There were 3, 6, and 14 indicator fungal OTUs for three sampling years (Table 2). In March of 2013, all the indicator species are putative ERM fungi, such as *Cryptosporiopsis ericae*^34^,^37^, *Oidiodendron maius*^22^,^37^,^38^, Myxotrichaceae 2 and three species of Dermateaceae (Helotiales). Indicators for March of 2014 were diverse, including putative ERM fungi (Helotiales 6, Helotiales 7, Helotiales 8, Hypocreales 1^35^,^39^,^40^, and Sebacinales 1), putative ECM fungi (*Tomentella* sp1 and *Tomentellopsis* sp1), as well as saprotrophic fungi (Malasseziales 1, *Penicillium herquei, Penicillium* sp3, *Penicillium* sp6 and *Penicillium* sp7).

There were 22 OTUs that exhibited significant preference to human disturbances (Table 3). The numbers of indicator species for OGF, SECI, SECII, and PLF were 7, 0, 2, and 13, respectively. The low number of indicator species in two secondary forests may be related to their similar abiotic and biotic factors to the old growth forest, and much higher number of indicator species in PLF may be due to the distinct abiotic and biotic factors in plantations. Four indicative fungal species in OGF belong to putative ERM fungi, i.e. Myxotrichaceae 1 and three OTUs of Herpotrichiellaceae (Herpotrichiellaceae 1, Herpotrichiellaceae 2 and *Cladophialophora chaetospira*). Of the indicator species for PLF, *Sebacina* sp1, *Sebacina* sp2, Herpotrichiellaceae 3 and Chaetothyriales 1 are from common ERM fungal orders, and *Diaporthe* sp1 and *Pestalotiopsis* sp1 are considered as plant pathogens.

### Factors affect fungal community associated with *V. carlesii* hair roots

Our results also showed that plant community, soil parameters, and geographic factors had significant effects on root-associated fungal community of *V. carlesii* (Table 4). For plant community, PC2, PC3 and species richness showed significant effects. The plant species that had significant contribution on PC2 and PC3 included the commercial species in PLF, i.e., *Cunninghamia lanceolata*, some dominant ECM plant species in subtropical forest, i.e., *Castanopsis eyrei* and *Lithocarpus glaber*, as well as some common ERM plant species, i.e., *R. ovatum*, *R. latoucheae* and *V. carlesii*. These results indicated that both dominant trees and common ERM neighbors may affect fungal community of *V. carlesii*. For soil parameters, soil organic carbon (SOC), soil total nitrogen (STN), soil total phosphorus (STP), and ammonium nitrogen (NH_4_^+^-N) had significant effects on fungal community of *V. carlesii*. Longitude and latitude are significant geographic factors, showing that geographic distribution of plots may have significant effects on fungal community of *V. carlesii.*

The Adonis results showed that forest type, year, longitude, and latitude had significant effects on fungal community of *V. carlesii* (Table 5). The effects of season and altitude were not significant.

## Discussion

### Diversity of total fungal OTUs associated with *V. carlesii* hair roots

Diverse fungal species were observed in hair roots of *V. carlesii* in the present study. These fungal species include typical ERM fungal orders such as Helotiales and Sebacinales, which have been observed in roots of many ericaceous plant species^1^,^33^,^34^,^37^,^38^,^41^,^42^. Some fungal families, such as Myxotrichaceae^37^,^39^, Herpotrichiellaceae^41^,^43^, Dermateceae^1^,^22^,^34^,^35^, and Sebacinaceae^6^,^43^,^44^ considered as ERM fungi, were frequently found in our results. Some putative ERM fungal taxa were detected in our study, such as *Lachnum*^1^, *Scytalidium*^40^, *Meliniomyces*^1^,^29^, *Gliocladium*^39^, *Cryptosporiopsis ericea*^1^, *Oidiodendron maius*^2^,^22^,^37^,^39^,^45^ and *Rhizoscyphus ericae*^22^,^45^,^46^. The latter two fungal species which were frequently cultured from ericaceous roots as typical ERM fungi, was less in our results, and coincidence with the results from Allen *et al.*^43^ and Bougoure and Cairney^47^ research, suggesting that these fungi were not dominants of ERM fungal communities in open ecosystems.

Some typical ECM fungal genera were also found in roots of *V. carlesii*, for example *Russula*, *Tomentella*, *Rhizopogon*, *Thelephora, Cenococcum.* Those fungal genera were also observed in roots of some other ericaceous plants^36^,^42^,^43^, indicating that they may form symbiotic structures with both ERM and ECM host plants. In subtropical forests of China, the dominant tree species of canopy are usually ECMF hosts (e.g. Fagaceae) and Ericaceae plants are dominant in the shrub layer. Common mycorrhizal networks formed between ECM and ERM hosts may be essential for species coexistence and ecosystem functioning in subtropical forests.

Besides of mycorrhizal fungi, DSE (e.g., *Phialocephala fortinii*^1^,^41^,^43^,^47^,^48^), saprobes^34^,^35^,^42^, pathogens^1^,^42^ and unidentified root-associated fungi were also present in hair roots of *V. carlesii*. DSE was found frequently co-exist in ericaceous roots^30^,^32^,^49^ in heathlands, forests and alpine ecosystems^50^ and showed stronger resistant to adverse conditions such as drought, repeated freezing and thawing^51^. Ecological functions of saprobes (such as *Penicillium* spp.) observed in our investigation are still unknown, while it has been documented that some typical saprophytic fungal genera were also observed in roots of ERM plants in previous studies, e.g., *Acremonium*^40^, *Capronia*^42^,^43^, *Myrothecium*^43^. One possible reason for the occurrences of saprobes is that these fungi may act as endophytes at some stages of their lifecycle and saprobes in soil at other stages. It have been found that putative ERM fungi (e.g. *Oidiodendron maius*^52^,^53^ and *R. ericae*^53^,^54^) could live as saprotrophs for a long time when their hosts were absent^55^.

Compared with traditional culture-based, isolated and morphological identified approaches, application of high-throughput sequencing makes a big progress in the studies of mycorrhizal and soil fungal diversity and their communities^23^,^24^,^56^,^57^,^58^,^59^,^60^, because it can generate unprecedented numbers of sequences, and even very rare and low-abundance organisms^61^,^62^,^63^ can be detected. We observed 5595 fungal OTUs in our study sites, while in researches using on isolation and culture or local database based ITS-RFLP (Internal Transcribed Spacer-Restriction Fragment Length Polymorphism), Zhang *et al.*^37^, Tian *et al.*^39^, Sun *et al.*^44^ found only 17, 12 and 35 fungal taxa, respectively. Helotiales and Sebacinales were usually found in the previous studies in ericaceous plants^44^. Our results using high-throughput sequencing not only found these fungal taxa, but also diverse fungal taxa in roots of *V. carlesii.* Diverse fungal taxa were also observed when studying other mycorrhizal fungal community using high-throughput sequencing. Buscardo *et al.*^60^ supposed that the number of ECMF taxa was eight-fold higher by using 454 pyrosequencing than by DGGE (Denaturing Gradient Gel Electrophoresis), and Oja Jane *et al.*^59^ obtained 5805 OTUs in orchid mycorrhizal symbionts by 454 pyrosequencing.

### Intra- and inter- annual dynamics of root- associated fungal communities

Seasonal changes of dominant fungal taxa in roots of *V. carlesii* have been found in the present study (Fig. 2B) and the ratio of Basidiomycota to Ascomycota is significantly correlated with monthly mean temperature (Fig. 5). In another study conducted in the typical Mediterranean climate regions of Australia, researchers also found significant seasonal variations of ERM fungi, in which ERM colonization and diversity were higher in colder and more humid winter^20^,^21^,^23^. Soil moisture has been suggested as a key factor in determining seasonal changes of ERM communities^24^. However, in the subtropical region of our study, the highest precipitation usually occurs from June to August and water might not always be a limiting factor in determining seasonal patterns of fungal community in roots of *V. carlesii.* The total and specific number of OTUs occurred in spring were more than in three other seasons, implying that the colder and more humid climate in our investigation region might be benefit to most fungi development.

The seasonal changes of common fungal taxa and the ratio of Basidiomycota to Ascomycota also imply that different fungal taxa may respond differently to seasonal environmental changes and interactions between fungal taxa may also change within a year. Seasonality has been reported in AM and ECM fungal communities^16^,^17^,^64^,^65^ and soil fungi^66^, and seasonal changes may be initialized from ecological factors such as temperature, moisture, vegetation-soil interactions and substrate availability changes.

OTU numbers in spring of 2012 were extremely higher than that in 2013 and 2014 (Fig. 3C) and the results of PCA showed significant difference in root associated fungal community between years (Fig. 4). When comparing the samples collected in spring from 2012 to 2014, obvious fluctuations of fungal communities were observed (in Figs 4 and S2). Most of Zhejing province including our study site experienced an extremely drought during the summer of 2013, which could affect fungal of 2014 spring. The distinct fungal community in spring of three years and more indicator species for the year 2014 could be partially due to the inter-annual shifts of climatic factors. The mechanisms underlying inter-annual shifts of root-associated fungal community may be quite complex, and changes in plant community, edaphic factors, and climatic parameters may be involved. For example, roots development and mycorrhizal infection may be affected by moisture regimes, which depend on the rainfall variations related with the El Niño or La Nina years in Australia^20^,^43^. Cairney & Ashford^20^ found that in El Niño year with summer drought, ERM colonization and amount of hair roots are positively correlated with soil moisture, while in La Nina year without summer drought, temperature was the determinant factor for ERM structures^20^. Therefore, limited factors on hair roots and ERM fungi development were different by seasons and years. Sometimes sampling time can also strongly affect the microbial community compositions^67^.

### Root associated fungal community along human disturbance gradients

All the forest types once covered by the same vegetation of subtropical forests, with dominant plant families of Fagaceae, Theaceae, Lauraceae, Pinaceae, Taxodiaceae, etc. Human disturbance extremely altered dominant species composition of plants, especially in *Cunninghamia lanceolata* plantation (Table S2).

Our results showed that human disturbances, especially the *C. lanceolata* plantation significantly changed community structure of root associated fungi of *V. carlesii.* The number of total and specific OTUs in PLF was much higher than those in two secondary forests with more DSE and pathogen species, and both of these fungal types were found often present in earlier stages of succession^27^,^68^,^69^ with less mycorrhizal fungi^30^. More proportion of ERM fungi co-existed in the fungal communities in OGF probably due to the colonization rate of mycorrhizal fungi would increase along with the soil development years^21^,^70^. Compared with plantations, secondary forests have more similar root-associated fungal communities with each other and closer to the old growth forest, which may be attributed to the relatively similar accompanying plant compositions and similar abiotic environmental parameters between old growth forests and secondary forests. The distinct root-associated fungal community of *V. carlesii* in PLF might be due to (1) the different mycorrhizal status of dominant plants between PLF and other forest types, since *C. lanceolata* is AM plants^71^ while the dominant plant family (Fagaceae) is ectomycorrhizal; (2) the differences in soil parameters between PLF and other three forest types; and (3) the spatial aggregation and isolation. Our results also showed that plant community, soil parameters, and geographic factors had significant effects on root-associated fungal community of *V. carlesii* (Table 4). Results from some previous studies also suggested that old forests and secondary forests were more similar in fungal communities though the disturbance patterns were different^24^,^32^,^72^, and they would be more and more similar in soils over time^7^,^10^,^11^.

Fungal diversity and its dynamic changes have been investigated after plant composition and abiotic factors varied^12^,^13^,^70^. Environmental changes make the same hosts have different fungal communities, for example, Bougoure *et al.*^73^ examined root-associated fungal communities of *Calluna vulgaris* along a heath to forest gradient in Scotland and found significant differences; and Hazard *et al.*^12^ also found that diversity of fungi associated with hair roots of *Vaccinium macrocarpon* in peatland of Ireland was affected by three different land use (bog, rough grazing and forest plantation). Human disturbance happened frequently in forest ecosystems, however, in some cases, even after over fifty years of conversion from land use^8^,^9^, or more than one century succession^70^, significant difference between older and earlier forest ecosystems would still be observed in microbial community structure. The effects of disturbance on the species and richness of soil and mycorrhizal fungi were also found in other studies on ECM, AM and soil fungal communities^29^,^70^,^74^. The responses of root-associated fungi to human disturbance gradients may depend much on the host plants, the ecosystems selected and the extent of disturbance gradients.

## Conclusions

Diverse fungal OTUs have been observed in hair-roots of *Vaccinium carlesii* using high-throughput sequencing, including putative ERM fungi, ECM fungi, common DSE, saprobes and pathogens. Dominant phyla are Ascomycota and Basidiomycota, and common classes are Leotiomycetes, Eurotiomycetes, Dothideomycetes, Sordariomycetes and Agaricomycetes. Intra- and inter- annual variability in root-associated fungal community of *V. carlesii* have been observed in the present study, and the ratio of Basidiomycota to Ascomycota is related to monthly mean temperature of the sampling month. Significant differences were found between different forest types along the disturbance gradient, and root-associated fungal communities of *V. carlesii* in two secondary forest types are similar with each other and are closer to that in old growth forests. Factors affecting root-associated fungal communities of *V. carlesii* include plant community composition, edaphic parameters, and geographic factors.

## Materials and Methods

### Study sites

The study site is located at Gutianshan National Nature Reserve (GNNR), Zhejiang Province in East China (29°10′19.4″N–29°17′41.4″N, 118°03′49.7″E–118°11′12.2″E). Annual mean temperature is 15.3 °C and annual precipitation ranges from 1793 to 1960 mm. Subtropical red soil with granite or deeply weathered granite as parent rock is the dominant soil type^75^. The typical vegetation in this region is subtropical evergreen broad-leaved forest^76^, with *Castanopsis eyrei* and *Schima superba* being the dominant canopy species, and *Vaccinium carlesii* is one of the common understory shrubs with abundant individuals in this area (Table S2).

Four types of forests with different disturbance history in the GNNR were studied in the present study: old growth forests (OGF), secondary forests with once cutting (SEC I), secondary forests with twice cutting (SEC II), and *Cunninghamia lanceolata* plantation (PLF). Within each type of forests, three 1-ha (100 m × 100 m) plots were randomly selected SEC I was clearly cut about 50 years ago, while SEC II was clearly cut about 50 years ago and then selectively cut about 20 years ago. PLF was planted about 20 year ago after clear cutting of secondary forests. Stands in both types of secondary forests and plantations have been undergoing natural recovery since last anthropogenic disturbances. OGF is undisturbed forests that did not experience tree-felling during the last 100 years and is generally located at the core zone of GNNR^77^.

### Sampling procedure

Hair roots of four individuals of *V. carlesii* were sampled in March, June, September, December of 2012 and March of 2013 and 2014 from each plot. In total, root samples from 288 individuals were collected (4 samples × 12 plots × 6 sampling times). In detail, the terminal portion of the finer roots (typical ericaceous “hair roots”) was retrieved from soils at four directions around the trunk of each *V. carlesii* individual. The roots were washed carefully after 1 h soaking in sterile water. Hair roots were then cut into 1 cm segments and 20 hair root segments were selected randomly from each *V. carlesii* individual sample. Each root segment was put into a centrifuge tube and preserved in 70% alcohol at −70 °C before DNA extraction. A 200 g soil sample was taken from the top 10 cm of soil adjacent to each plant sampled for elemental analyses.

### DNA extraction

DNA was extracted from hair roots of *V. carlesii* following the protocol of DNA secure Plant Kit (TIANGEN Biotech Co. Ltd), with slight modifications. Hair root segments were put into a sterile centrifuge tube containing 20 μl 2× CTAB extraction buffer solution and ground with a plastic pestle on ice. Samples were warmed at 65 °C for 1 h in 630 μl aliquot of 2× CTAB extraction buffer solution, and then shaken for 10 min. Aliquot of chloroform/isoamyl alcohol (24:1) was added followed by twice centrifugation at 13,201 × g for 8 min at room temperature. The supernatant was precipitated with 100% alcohol at 4 °C for 1 h followed by centrifugation at 17,968 × g for 8 min. DNA precipitate was washed twice using 70% ethanol, dried in a vacuum desiccator, and dissolved in 30 μl sterile ddH_2_O at 4 °C. DNA samples were stored at −22 °C prior to downstream analyses.

### PCR amplifications

The ITS1F-ITS2 region of fungi were amplified by PCR (95 °C for 2 min, followed by 25 cycles at 95 °C for 30 s, 55 °C for 40 s, and 72 °C for 50 s and a final extension at 72 °C for 5 min) using primers ITS1F 5′-barcode-CTTGGT CATTTAGAGGAAGTAA-3′ and ITS2 5′-GCTGCGTTCTTCATCGATGC-3′, where barcode is an eight-base sequence unique to each sample. PCR reactions were performed in triplicate 20 μl mixture containing 4 μl of 5× FastPfu Buffer, 2 μl of 2.5 mM dNTPs, 0.8 μl of each primer (5 μM), 0.4 μl of FastPfu Polymerase, and 10 ng of template DNA.

### Illumina MiSeq sequencing

Amplicons were extracted from 2% agarose gels and purified using the AxyPrep DNA Gel Extraction Kit (Axygen Biosciences, Union City, CA, US) following the manufacturer’s instructions and quantified using QuantiFluor™-ST (Promega, U.S.). Purified amplicons were pooled in equimolar and paired-end sequenced (2 × 250) on an Illumina MiSeq platform adopting the standard protocols.

### Bioinformatic analysis

Raw fastq files were demultiplexed andquality-filtered using QIIME (ver 1.7) with the following criteria: (i) the reads were truncated at any site receiving an average quality score <20 over a 10 bp sliding window, discarding the truncated reads that were shorter than 50 bp; (ii) exact barcode matching, 2 nucleotide mismatch in primer matching, reads containing ambiguous characters were removed; (iii) only sequences that overlapped by longer than 10 bp were assembled according to their overlap sequence. Reads that could not be assembled were discarded.

Open reference OTU picking was done with pick_open_reference_otus.py using the default uclust method and ITS 12-11 dataset (97% similarity cutoff was used, alpha release, download from web site of QIIME http://qiime.org/home_static/dataFiles.html), and singletons were removed during OTU picking. The phylogenetic affiliation of each sequence was analyzed by RDP Classifier (http://rdp.cme.msu.edu/) against ITS-12-11 dataset using confidence threshold of 80%. Single_rarefaction.py in Qiime was used to generate OTU table with even reads of 10,000 in each root sample.

### Statistical analysis

One-way ANOVA (with post hoc comparisons using Duncan’s test) was carried out to test the difference of individual density and mean DBH per individual of *V. carlesii* between four forest types using SPSS (ver. 16.0, SPSS Inc.). Indicator species analysis was performed using out_category_significance.py in Qiime to determine the indicator fungal species for different forest types, seasons, and years. “Envfit” function was used to identify the main factors influencing root-associated fungal community. Principal Components Analysis (PCA) was performed using “rda” function in the R package “vegan” and the first three components (PC1, PC2, and PC3) were used as plant parameters in “envfit”. “Adonis” function in the R package “vegan” was used to evaluate the impacts of forest type, year, season, and geographic factors on root-associated fungal community of *Vaccinium carlesii.*

### Ethics Statement

No specific permits were required for the described field studies. The study sites are not privately-owned or protected in any way, and the field studies did not involve endangered or protected species.

## Additional Information

**How to cite this article**: Zhang, Y. *et al.* Root-associated fungi of *Vaccinium carlesii* in subtropical forests of China: intra- and inter-annual variability and impacts of human disturbances. *Sci. Rep.* **6**, 22399; doi: 10.1038/srep22399 (2016).

## Supplementary Material

Supplementary Information

## Figures and Tables

**Table 1:** Indicator fungal species associated with hair roots of *V. carlesii* in four sampling seasons.

Index	Identified name	Group	*P*
1	Dermateaceae 1	Spring	0.019
2	Helotiales 2	Spring	0.025
3	Helotiales 3	Spring	0.016
4	Helotiales 4	Spring	0.003
5	Helotiales 5	Spring	0.022
6	Herpotrichiellaceae 4	Spring	0.039
7	Leotiomycetes 1	Spring	0.011
8	*Mortierella* sp1	Spring	0.008
9	*Parmelia* sp1	Spring	0.021
10	*Penicillium* sp3	Spring	0.043
11	*Penicillium spinulosum*	Spring	0.048
12	*Phialocephala fortinii*	Spring	0.044
13	*Pochonia bulbillosa*	Spring	0.013
14	Thelephoraceae 1	Spring	0.011
15	unidentified fungus 2	Spring	0.011
16	Eurotiomycetes 1	Summer	0.021
17	*Trichoderma* sp1	Summer	0.02
18	Ascomycota 4	Autumn	0.043
19	Ascomycota 5	Autumn	0.002
20	Agaricomycetes 1	Autumn	0.021
21	Basidiomycota 1	Autumn	0.031
22	Herpotrichiellaceae 5	Autumn	0.001
23	*Paecilomyces* sp1	Autumn	0.018
24	*Penicillium* sp4	Autumn	0.005
25	*Penicillium* sp5	Autumn	0.005
26	Thelephoraceae 2	Winter	0.049
27	Trechisporales 1	Winter	0.049

**Table 2:** Indicator fungal species associated with hair roots of *Vaccinium carlesii* for spring of different sampling years.

Index	Identified name	Group	*P*
1	Eurotiomycetes 2	2012	0.0016
2	unidentified fungus 3	2012	0.0006
3	unidentified fungus 4	2012	0.0074
4	*Cryptosporiopsis ericae*	2013	0.0052
5	Dermateaceae 2	2013	0.0004
6	Dermateaceae 3	2013	0.0037
7	Dermateaceae 4	2013	0.0047
8	Myxotrichaceae 2	2013	0.0005
9	Oidiodendron maius	2013	<0.0001
11	Debaryomyces sp1	2014	0.0007
10	Helotiales 6	2014	0.0065
12	Helotiales 7	2014	0.0083
13	Helotiales 8	2014	0.0022
14	Hypocreales 1	2014	0.0077
15	Malasseziales 1	2014	0.0001
16	*Penicillium herquei*	2014	0.0068
17	*Penicillium* sp3	2014	0.0023
18	*Penicillium* sp6	2014	0.0001
19	*Penicillium* sp7	2014	0.0018
20	Sebacinales 1	2014	0.0059
21	*Tomentella* sp1	2014	0.0044
22	*Tomentellopsis* sp1	2014	0.0015
23	unidentified fungus 5	2014	0.0096

**Table 3:** Indicator fungal species associated with hair roots of *V. carlesii* in forests with different human disturbances.

Index	Identified name	Group	*P*
1	Ascomycota 1	OGF	0.0002
2	Myxotrichaceae 1	OGF	0.0001
3	Herpotrichiellaceae 1	OGF	0.0001
4	Herpotrichiellaceae 2	OGF	0.0018
5	*Cladophialophora chaetospira*	OGF	0.0002
6	Penicillium sp1	OGF	0.0020
7	unidentified fungus 1	OGF	0.0025
8	Sebacinaceae 1	SECII	0.0009
9	Ascomycota 2	SECII	0.0017
10	Helotiales 1	PLF	0.0008
11	Penicillium sp2	PLF	0.0015
12	Diaporthe sp1	PLF	0.0048
13	Trichoderma sp1	PLF	0.0028
14	Neonectria sp1	PLF	0.0055
15	Pestalotiopsis sp1	PLF	0.0099
16	Ascomycota 3	PLF	0.0014
17	Clitopilus prunulus	PLF	0.0036
18	Sebacina sp1	PLF	<0.0001
19	Sebacina sp2	PLF	<0.0001
20	Herpotrichiellaceae 3	PLF	<0.0001
21	Sordariomycetes 1	PLF	0.0024
22	Chaetothyriales 1	PLF	0.0044

**Table 4:** Correlations of microbial community composition with plant community, edaphic and geographic factors.

	RDA1	RDA2	r^2^	*P*
Plant community				
PC1	−0.774	0.633	0.005	0.845
PC2	0.980	0.201	0.188	<0.001
PC3	−0.443	0.896	0.186	0.003
Species richness	0.830	−0.558	0.082	0.050
TABH	−0.957	0.292	0.065	0.094
Edaphic parameters				
SOC	0.829	−0.560	0.129	0.012
STN	0.839	−0.544	0.114	0.019
STP	0.303	−0.953	0.100	0.025
NH4^+^-N	0.779	−0.627	0.142	0.006
NO3^−^-N	0.729	0.684	0.060	0.118
AP	0.865	−0.501	0.029	0.362
pH	−0.589	−0.808	0.014	0.628
Geographic factors				
Longitude	−0.667	−0.745	0.154	0.003
Latitude	0.507	0.862	0.215	<0.001
Altitude	−0.392	0.920	0.060	0.116

Abbr.s: TABH, total area at breast height; PC1, PC2 and PC3, the first three principal components of plant community; SOC: soil organic carbon, STN: soil total nitrogen content, STP: soil total phosphorus content, NH_4_^+^-N: ammonium nitrogen, NO_3_-N: nitrate nitrogen, AP: Available phosphorus.

**Table 5:** Adonis results showing effects of different factors on root-associated fungal community of *Vaccinium carlesii.*

	df	SS	MS	*F*	*P*
Forest type	3	2.650	0.883	3.291	0.001
Year	2	2.769	1.385	5.159	0.001
Season	3	0.922	0.307	1.145	0.204
Longitude	1	0.441	0.441	1.645	0.046
Latitude	1	0.560	0.560	2.086	0.007
Altitude	1	0.369	0.369	1.376	0.096
Residuals	60	16.103	0.268	0.676	
