# Supplementary material for: Root-associated fungi of Vaccinium carlesii in subtropical forests of China: intra- and inter-annual variability and impacts of human disturbances
Source: Sci Rep. 2016 Mar 1;6:22399. doi: 10.1038/srep22399 (PMC4772160; doi:10.1038/srep22399)
Supplement: Supplementary Information [file srep22399-s1.doc]

Supplementary materials

Root-associated fungi of *Vaccinium carlesii* in subtropical forests of China: intra- and inter-annual variability and impacts of human disturbances

Yanhua ZHANG^1^, Jian NI^1^, Fangping TANG^1^, Kequan PEI^2^, Yiqi LUO^3^, Lifen JIANG^3^, Lifu SUN^1,3*^, Yu LIANG^2*^


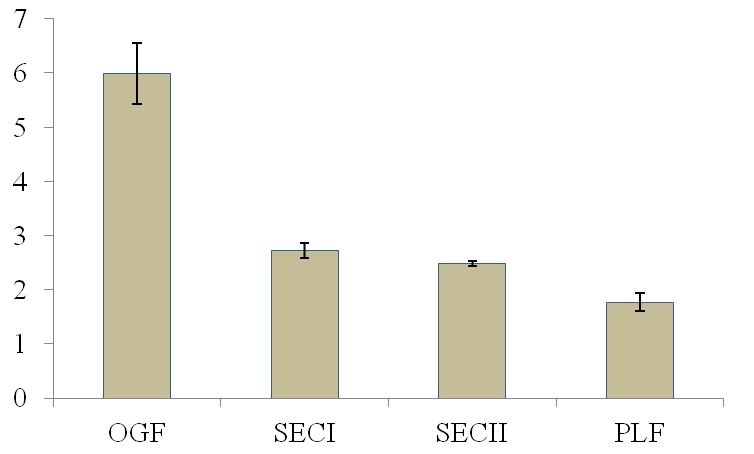

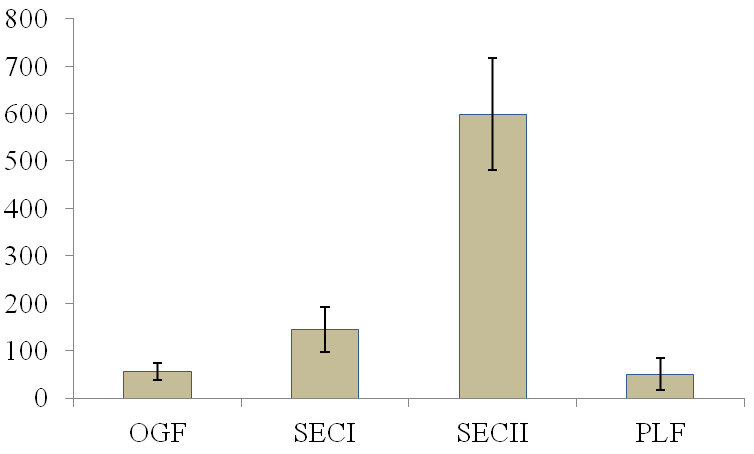


DBH (cm)

Individual density (No. per ha)

Forest types

Fig.S1 Mean DBH and individual density of *V. carlesii* in forests with different human disturbances

Mar-12 Jun-12 Sep-12 Dec-12 Mar-13 Mar-14


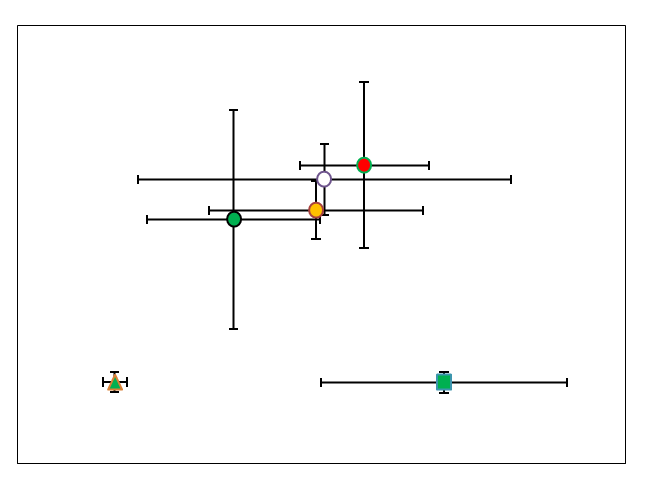

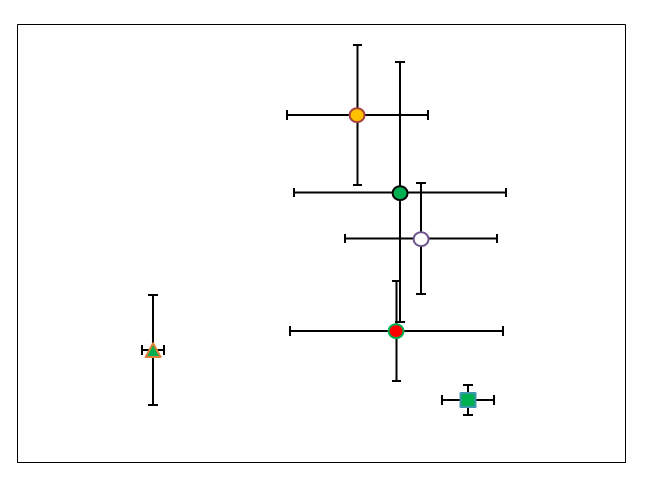

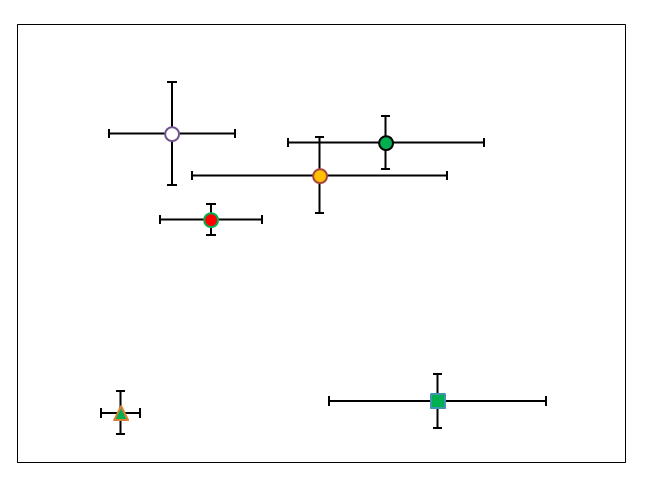

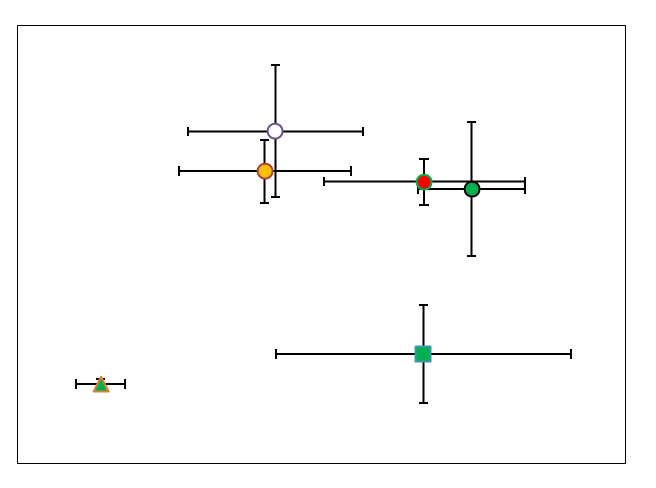


OGF

SECI

PC2 (8.4%)

SECII

PLF

PC1 (18.2%)

Fig. S2 Principal Component Analysis (PCA) of root-associated fungi of *V. carlesii* in different sampling seasons in forests with different human disturbances.


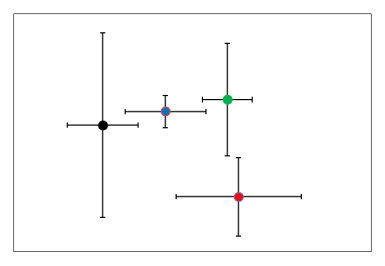

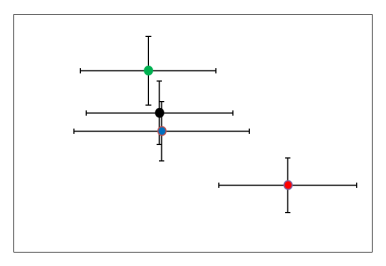

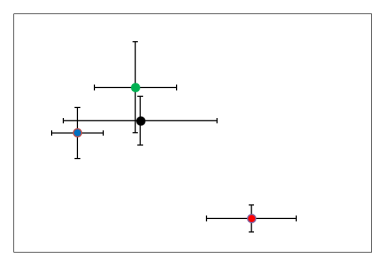

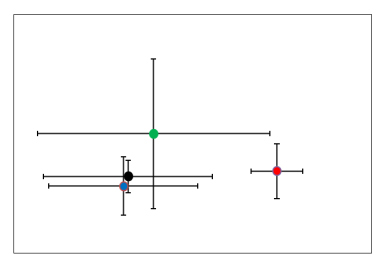

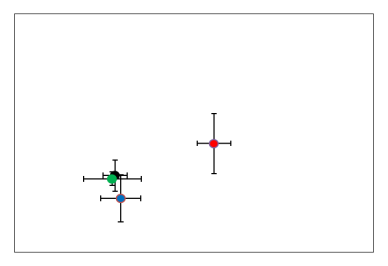

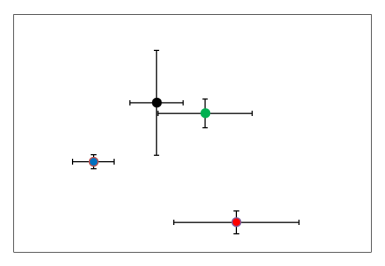


PC2 (8.4%)

Fig. S3 Principal Component Analysis (PCA) of root-associated fungi of *V. carlesii* in forests with different human disturbances in different sampling seasons.

Spring-14

Spring-13

Winter-12

Autumn-12

Summer-12

Spring-12

PC1 (18.2%)


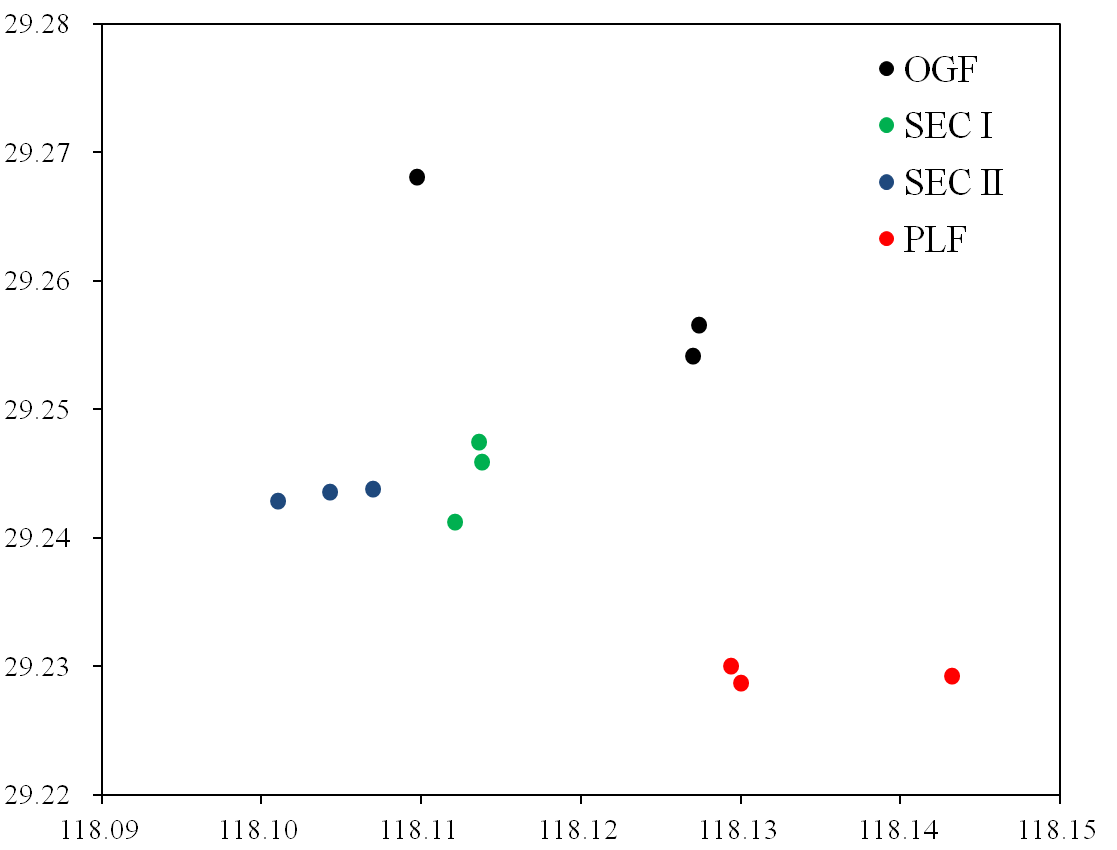


Latitude (ºN)

Fig. S4 Distribution of sampling plots.

Longitude (ºE)

Table S1. Soil parameters of four forest types in the present study, i.e., old growth forest (OGF), secondary forest I (SEC I), secondary forest II (SEC II), and plantation (PLF).

| Forest type | SOC  (g/kg) | STN  (g/kg) | STP  (g/kg) | NH_4_^+^-N  (mg/kg) | NO_3_^-^-N  (mg/kg) | AP  (mg/kg) | pH |
| --- | --- | --- | --- | --- | --- | --- | --- |
| OGF | 66.7±10.4a | 3.18±0.51a | 0.19±0.06a | 41.3±5.7a | 2.1±0.6b | 6.2±1.2a | 4.77±0.03a |
| SEC I | 38.8±4.2b | 1.97±0.19b | 0.17±0.02a | 30.3±2.4a | 3.2±0.1ab | 4.3±0.9ab | 4.77±0.02a |
| SEC II | 39.9±1.4b | 1.89±0.10b | 0.14±0.01a | 28.6±0.7a | 3.7±0.3a | 2.9±0.2b | 4.73±0.02a |
| PLF | 43.1±2.5b | 2.12±0.16b | 0.20±0.01a | 33.1±4.5a | 2.5±0.6ab | 4.3±0.2ab | 4.75±0.02a |

SOC: soil organic carbon

STN: soil total nitrogen content

STP: soil total phosphorus content

NH_4_^+^-N: ammonium nitrogen

NO_3_^-^-N: nitrate nitrogen

AP: Available phosphorus

| Plant species | Family | OGF | SEC I | SEC II | PLF |
| --- | --- | --- | --- | --- | --- |
| *Rhododendron ovatum* | Ericaceae | 8.59 | 5.71 | 5.08 | 2.19 |
| *Rhododendron latoucheae* | Ericaceae | 8.07 | 0.73 | 4.57 | 0.33 |
| *Castanopsis eyrei* | Fagaceae | 7.13 | 3.75 | 12.32 | 0.44 |
| *Camellia fraternal* | Theaceae | 6.57 | 3.82 | 1.71 | 0.81 |
| *Eurya muricata* | Pentaphylacaceae | 4.88 | 5.02 | 2.80 | 5.71 |
| *Distylium myricoides* | Hamamelidaceae | 4.62 | 0.00 | 0.85 | 0.03 |
| *Eurya rubiginosa* | Pentaphylacaceae | 4.55 | 0.53 | 1.99 | 0.41 |
| *Camellia chekiang-oleosa* | Theaceae | 4.45 | 0.00 | 0.01 | 0.28 |
| *Cyclobalanopsis glauca* | Fagaceae | 3.70 | 5.73 | 3.22 | 0.53 |
| *Corylopsis glandulifera* | Hamamelidaceae | 3.46 | 0.02 | 0.00 | 0.00 |
| *Cinnamomum subavenium* | Lauraceae | 3.45 | 0.14 | 0.28 | 0.01 |
| *Chimonanthus salicifolius* | Calycanthaceae | 3.30 | 0.00 | 0.13 | 5.74 |
| *Schima superb* | Theaceae | 2.98 | 5.56 | 2.76 | 1.14 |
| *Neolitsea aurata* | Lauraceae | 2.48 | 0.09 | 2.45 | 0.05 |
| *Symplocos anomala* | Symplocaceae | 2.20 | 0.20 | 0.43 | 0.01 |
| *Vaccinium carlesii* | Ericaceae | 1.53 | 2.98 | 5.79 | 0.76 |
| *Loropetalum chinensis* | Hamamelidaceae | 1.42 | 10.87 | 4.75 | 5.72 |
| *Vaccinium mandarimorum* | Ericaceae | 1.08 | 2.04 | 0.26 | 0.46 |
| *Pinus massoniana* | Pinaceae | 0.76 | 3.45 | 1.66 | 1.41 |
| *Itea* *omeiensis* | Saxifragaceae | 0.66 | 3.33 | 2.22 | 1.39 |
| *Vaccinium bracteatum* | Ericaceae | 0.63 | 2.21 | 1.55 | 0.32 |
| *Lithocarpus glaber* | Fagaceae | 0.56 | 7.76 | 6.64 | 2.98 |
| *Rhaphiolepis indica* | Rosaceae | 0.55 | 0.35 | 2.10 | 0.07 |
| *Rhododendron simsii* | Ericaceae | 0.49 | 2.30 | 1.31 | 1.60 |
| *Toxicodendron succedaneum* | Anacardiaceae | 0.29 | 0.71 | 2.56 | 0.04 |
| *Syzygium buxifolirm* | Myrtaceae | 0.21 | 0.87 | 4.27 | 0.18 |
| *Adinandra millettii* | Pentaphylacaceae | 0.07 | 3.43 | 3.65 | 1.44 |
| *Castanopsis carlesii* | Fagaceae | 0.06 | 4.37 | 4.01 | 0.01 |
| *Cunninghamia lanceolata* | Cupressaceae | 0.01 | 0.51 | 0.10 | 52.89 |
| *Machilus grijsii* | Lauraceae | 0.00 | 3.34 | 1.51 | 0.38 |

Table S2. Relative abundances (%) of common woody plant species (>2%) in four forest types of the study site
